# Sensing the Stress: A Role for the UPR^mt^ and UPR^am^ in the Quality Control of Mitochondria

**DOI:** 10.3389/fcell.2018.00031

**Published:** 2018-03-28

**Authors:** Sylvie Callegari, Sven Dennerlein

**Affiliations:** Department of Cellular Biochemistry, University Medical Center Göttingen, Göttingen, Germany

**Keywords:** mitochondria, UPR signaling pathways, mitochondrial translation, mitochondrial signaling, cytochrome c oxidase

## Abstract

Mitochondria exist as compartmentalized units, surrounded by a selectively permeable double membrane. Within is contained the mitochondrial genome and protein synthesis machinery, required for the synthesis of OXPHOS components and ultimately, ATP production. Despite their physical barrier, mitochondria are tightly integrated into the cellular environment. A constant flow of information must be maintained to and from the mitochondria and the nucleus, to ensure mitochondria are amenable to cell metabolic requirements and also to feedback on their functional state. This review highlights the pathways by which mitochondrial stress is signaled to the nucleus, with a particular focus on the mitochondrial unfolded protein response (UPR^mt^) and the unfolded protein response activated by the mistargeting of proteins (UPR^am^). Although these pathways were originally discovered to alleviate proteotoxic stress from the accumulation of mitochondrial-targeted proteins that are misfolded or unimported, we review recent findings indicating that the UPR^mt^ can also sense defects in mitochondrial translation. We further discuss the regulation of OXPHOS assembly and speculate on a possible role for mitochondrial stress pathways in sensing OXPHOS biogenesis.

## Introduction

The eukaryotic cell is composed of different organelles, each fulfilling a variety of specific functions. In the last decades, organelles, such as the nucleus, the ER and mitochondria, have been extensively investigated as separate units. Nevertheless, emerging evidence describes how organelles are connected and how they communicate with each other. Mitochondria, which are the main cellular energy producers in the form of ATP, have attracted a particular focus. However, mitochondria are also involved in many other essential cellular processes such as Ca^2+^ homeostasis, Fe-S cluster biogenesis or the synthesis of critical metabolites, such as NADH/NAD^+^ and succinate/α-ketoglutarate (McBride et al., 2006). Hence, mitochondria are now perceived as key cellular signaling organelles. In fact, they physically interact with the majority of membrane bound organelles within the cell (Eisenberg-Bord et al., 2016; Valm et al., 2017). The most well studied of these contact sites is between the mitochondria and the ER (Kornmann et al., 2009; Elbaz-Alon et al., 2015; Lewis et al., 2016; Cohen et al., 2017), but mitochondria are also in constant exchange with other organelles such as lysosomes (Raimundo, 2014; Diogo et al., 2017), lipid droplets (Nguyen et al., 2017), and peroxisomes (Sugiura et al., 2017). Consequently, many signaling pathways triggered by mitochondria during physiological or pathological situations have been identified, which impact fundamental cellular processes such as autophagy, cell division, cell differentiation, or anti-viral signaling (Liu and Butow, 2006; Koshiba, 2013; Xu et al., 2013). Hence, many initiated stresses can provoke the activation of mitochondrial stress responses and many fundamental aspects regarding the molecular function of involved factors remain unclear. Specifically, it remains unclear how mitochondrial signals are transported and how and where those signals originate. We review recent findings on the mitochondrial initiated stress response pathways of the UPR^mt^ (the mitochondrial unfolded protein response) and UPR^am^ (the unfolded protein response activated by mistargeting of proteins) within the context of mitochondrial translation and impaired OXPHOS assembly. Therefore, first we will introduce the UPR^mt^ and UPR^am^ pathways. In the following sections we will discuss these pathways with a specific focus on how they could originate by reduced mitochondrial translation and disturbed OXPHOS biogenesis, taking cytochrome *c* oxidase as an example.

## Dealing with proteotoxic stress: the UPR^mt^ and UPR^am^

The human mitochondrial genome contains more than 1300 proteins (Calvo et al., 2016). While only 13 proteins are encoded within mitochondria, the majority (>99%) are nuclear-encoded, synthesized in the cytosol and are imported into the organelle. The transport of these precursor proteins is facilitated by various import machineries, excellently summarized by two recent review articles ofWasilewski et al. (2017) and Wiedemann and Pfanner (2017). As a consequence, mitochondria receive a constant influx of proteins that need to be matured and assembled into functional complexes. To functionally integrate mitochondria into the cellular network, signaling pathways are required that monitor mitochondrial fitness and enable a coordination of mitochondrial function with cellular demands. A major signaling route occurs between mitochondria and the nucleus (Wasilewski et al., 2017; Melber and Haynes, 2018). This is particularly important since the accumulation of unassembled precursor proteins inside or outside mitochondria leads to proteotoxic stress and eventually to cell death (Ryan and Hoogenraad, 2007; Topf et al., 2016; Wasilewski et al., 2017). Two major signaling pathways have been identified that monitor the precise and timely delivery of cytosolic precursors to the mitochondria; the mitochondrial unfolded protein response (UPR^mt^) and the unfolded protein response activated by mistargeting of proteins (UPR^am^).

### The proteotoxic stress induced cascade originating within mitochondria—UPR^mt^

**Figure 1:**
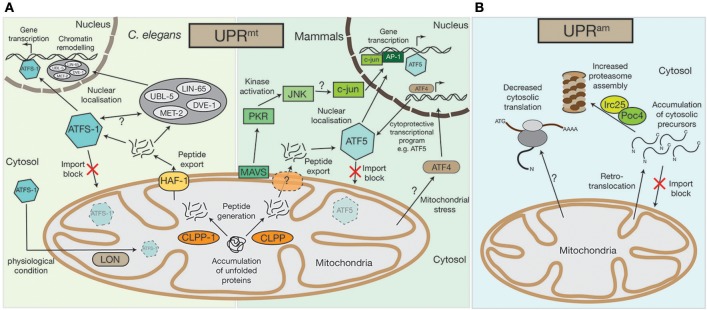
Mitochondrial UPR^mt^ and UPR^am^ stress response pathways. **(A)** An accumulation of unfolded proteins inside the mitochondrial matrix triggers the UPR^mt^ in both mammals and in nematodes. Accumulated proteins are likely processed by the CLPP protease and exported out of the mitochondria, a process that in *C.elegans* requires the HAF-1 protein. While under physiological conditions ATFS-1 gets imported into mitochondria and constitutively degraded by the AAA^+^-protease LON, exported peptides activate the transcription factor ATFS-1/ATF5 in the cytosol, which translocates to the nucleus to alter the cell's transcriptional program, particularly affecting the transcription of mitochondrial proteins. In *C.elegans* the UBL-5, LIN-65, and MET-2 proteins also translocate to the nucleus upon UPR^mt^ activation where they facilitate the binding of transcription factors, ATFS-1 and DVE-1, by chromatin remodeling. A second UPR^mt^ pathway has also been observed in mammals, whereby UPR^mt^ activation is sensed by the mitochondrial antiviral signaling protein MAVS by an unknown mechanism, which then triggers the activation of PKR, which in turn phosphorylates the c-Jun N-terminal kinase, JNK. JNK activates c-jun (also by an unknown mechanism), which translocates to the nucleus and, together with AP-1, alters gene transcription. The bZIP transcription factor AFT4 has also been linked to mitochondrial stress response pathways. Localized to the nucleus, ATF4 activates a complex cytoprotective transcriptional program, e.g., by transcriptional control of ATF5. **(B)** In lower eukaryotes, the accumulation of cytosolic precursors from either a block in mitochondrial import, from mislocalization, or from retrotranslocation out of the mitochondrial intermembrane space, can instigate the UPR^am^. This accumulation enhances activity of the Irc25/Poc4 chaperone complex, which is required for assembly of the proteasome. Increased proteasome assembly causes the rapid degradation of accumulated proteins. In an independent stress response mechanism observed in mammals, there is a decrease in cytosolic translation, which is coupled to increased proteasome activity, but the molecular mechanisms underlying this response remain to be defined.

The accumulation of misfolded or damaged proteins within the mitochondria can incite a range of proteotoxic stresses. For example, excess OXPHOS complex constituents leads to the generation of harmful sub-complexes, resulting in loss of membrane potential, or oxidative stress in the form of ROS production (Fernández-Vizarra et al., 2009; Fox, 2012; Soto et al., 2012; Timón-Gómez et al., 2017). One considerable possibility for the accumulation of non-assembled OXPHOS subunits could be defects in mitochondrial translation (see section Proteotoxic signaling cascades can be activated by defects in mitochondrial translation). One of the first lines of defense against such mitochondrial perturbations is the activation of the UPR^mt^ pathway, which is, besides defects in mitochondrial translation or OXPHOS biogenesis, responding to various mitochondrial stresses. The UPR^mt^ represents a conserved pathway between nematodes, flies, and mammals (Ryan and Hoogenraad, 2007; Quirós et al., 2016; Topf et al., 2016; Figure 1A). It is assumed that in nematodes, damaged or unassembled proteins are degraded by the AAA^+^-matrix protease CLPP-1, which would lead to the accumulation of peptides within the mitochondrial matrix (Haynes et al., 2007). However, if CLPP-1 exclusively degrades damaged or unassembled proteins is not clear, but all CLPP-1 generated peptides are derived from mitochondrial proteins. The transport of these peptides to the cytosol by the ABC transporter HAF-1 initiates signaling cascades outside mitochondria (Haynes et al., 2010). The stress activated bZIP transcription factor ATFS-1 seems to play a key role during these processes. In standard physiological conditions, ATFS-1 is localized to the mitochondrial matrix, where it is constitutively degraded by the AAA^+^-protease LON (Nargund et al., 2012). However, upon loss of import efficiency, such as during UPR^mt^ activation, ATFS-1 accumulates in the cytosol and, as it has a nuclear localization signal, it is relocalized to the nucleus where it acts as a transcriptional regulator (Nargund et al., 2012, 2015). ATFS-1 controls the expression of over 500 genes that impact several cellular processes (Nargund et al., 2012, 2015; Lin et al., 2016; Melber and Haynes, 2018). Among them are immune regulators [e.g., the antibacterial factor-related peptide 2 (Abf-2) (Nargund et al., 2012), metabolic enzymes [e.g., glutaminase (Nargund et al., 2015)] or additional transcription factors, such as the bZIP transcription factor skinhead-1 (Skn-1) (Nargund et al., 2012, 2015).

Additionally, multiple mitochondrial encoding genes, influencing the synthesis of proteotoxic stress related proteins (e.g., the 60 kDa heat shock protein HSP60 or the 70 kDa heat shock protein HSP70), as well as proteins of the oxidative phosphorylation machinery and TCA enzymes are targeted by ATFS-1 during mitochondrial stress (Nargund et al., 2015; Melber and Haynes, 2018). How ATFS-1 controls expression is not clear, however, studies in nematodes have shown that the homeobox transcription factor DVE-1, its cofactor the Ubiquitin-like protein UBL-5, the nuclear co-factor LIN-65, and the histone methyltransferase MET-2 are all involved (Tian et al., 2016a). These proteins are usually localized to the cytosol, but are recruited to the nucleus upon activation of the UPR^mt^ (Benedetti et al., 2006; Haynes et al., 2007; Tian et al., 2016a,b). LIN-65, together with MET-2, actively remodel chromatin structures, likely to enable binding of transcription factors (Merkwirth et al., 2016; Tian et al., 2016a,b). The transcription factors DVE-1 and ATFS-1 can then bind to the reorganized chromatin where, in a cascade of parallel pathways, they reprogram cell expression (Tian et al., 2016a).

Recently, the human homolog of ATFS-1, activating transcription factor 5 (ATF5), was identified (Fiorese et al., 2016). Similarly to ATFS-1, ATF5 also localizes to the nucleus upon UPR^mt^ activation. ATF5 has been found to be increased in patients with mitochondrial disorders (Endo et al., 2009; Tyynismaa et al., 2010; Torres-Peraza et al., 2013; Yap et al., 2016) and has an anti-apoptotic function, since it increases the expression of the B-cell lymphoma protein (BCL-2), which antagonizes apoptosis (Persengiev, 2002; Fiorese et al., 2016). Recently, another bZIP transcription factor, the activating transcription factor 4 (ATF4), was also linked to the mitochondrial stress response (Martínez-Reyes et al., 2012; Quirós et al., 2017; Melber and Haynes, 2018). Although, the exact relationship between ATF5 and ATF4 remains unclear, ATF4 has been postulated as ATF5 transcription factor (Melber and Haynes, 2018). Interestingly, a recent publication by Quirós et al. (2017) implied that ATF4, in contrast to ATF5, does not trigger the UPR^mt^ directly, but instead elicits a cytoprotective transcriptional program, which is part of a more general cell stress response known as the integrated stress response (ISR).

However, UPR^mt^ activation between nematodes and mammals seems to be conserved to a large extent, e.g., the Clp protease, CLPX, also induces the UPR^mt^ in mammals (Al-Furoukh et al., 2015), but a second, kinase regulated, stress response mechanism seems to exist in human cells that has yet to be detected in nematodes. Within this pathway, the protein kinase R (PKR), which is likely activated by the outer mitochondrial membrane antiviral signaling protein MAVS, phosphorylates the c-Jun N-terminal kinase JNK2 (Rath et al., 2012; Jacobs and Coyne, 2013). Next, JNK2 activates c-Jun, a component of the transcription factor AP-1, which then initiates a nuclear transcriptional response (Horibe and Hoogenraad, 2007; Rath et al., 2012).

However, our understanding of the UPR^mt^ pathways in nematodes and mammals is far from complete. Until now, we do not understand how peptides that are transported by HAF-1 from the mitochondrial matrix to the cytosol activate the UPR^mt^. Nor is it clear whether there are specific peptides that are required for UPR^mt^ activation, or what triggers the differential localization of ATFS-1/ATF5 from mitochondria to the nucleus. Furthermore, the mechanism that provokes the translocation of DVE-1, UBL-5, LIN-65, and MET-2 to the nucleus following initiation of the UPR^mt^ remains elusive. Similarly, for the human UPR^mt^ kinase activated system, the activation of the PKR kinase by MAVS remains enigmatic and requires further investigation.

### Stress induced signaling during protein mislocalization—UPR^am^

The second major signal initiation pathway during mitochondrial impairment has only been verified in lower eukaryotes, but there are some indications that similar pathways exist in mammalian cells (Papa and Germain, 2011; Wrobel et al., 2015; Wasilewski et al., 2017). Dysfunction of the mitochondrial import machinery eventually leads to cell death. However, if the import of cytosolic synthesized precursor proteins is only mildly impaired, a cytosolic protective program is activated. This “UPR^mt^ activated by mistargeting of proteins” (UPR^am^), involves the activation of the cytosolic proteasome (Wrobel et al., 2015) (Figure 1B). An increase of mitochondrial precursor proteins in the cytosol triggers the UPR^am^, leading to increased proteasome assembly by the enhanced activity of the proteasome assembly factors Irc25 and Poc4, which degrades excess proteins (Wrobel et al., 2015). The UPR^am^ protective stress response pathway seems not to be specific for a defined subset of precursor proteins, but rather represents a general mitochondrial dysfunction monitoring mechanism. Interestingly, it has been suggested that the UPR^am^ can also be activated by peptides and proteins that back-slide from the mitochondrial intermembrane space to the cytosol (Bragoszewski et al., 2015; Wasilewski et al., 2017). This indicates that the UPR^mt^ and UPR^am^ are either activated simultaneously, or that the presence of former mitochondrial matrix localized peptides induces the UPR^mt^, while retrotransported intermembrane space proteins activate the UPR^am^.

As mentioned, the UPR^am^ pathway has not been identified in higher eukaryotes, but an increase in proteasomal activity has been observed following proteotoxic stress (Papa and Germain, 2014). UPR^mt^ activation in invertebrates can decrease cytosolic translation, which has not been demonstrated in mammalian cells, yet. However, since cytosolic translation decreases upon mitochondrial dysfunction regardless of an accumulation of mitochondrial precursor proteins within the cytosol (Wang and Chen, 2015; Wrobel et al., 2015; Topf et al., 2016) it is tempting to speculate that besides the UPR^mt^ or UPR^am^ other stress response pathways are present in mammalian cells that can influence cytosolic translation. This decrease in cytosolic translation is likely elicited by a reduction of cytosolic 80S ribosomes, due to the reduced export of the 60S subunits from the nucleus (in yeast mediated by the nucleolar GTP binding protein, Nog2) (Wasilewski et al., 2017).

In conclusion, we are only beginning to shed light into the complex mechanisms of mitochondrial stress response pathways and it will be a challenge for the next decade to explore in detail how these signaling pathways are connected to aging processes and human disorders.

## Proteotoxic signaling cascades can be activated by defects in mitochondrial translation

The link between dysfunctional mitochondrial translation and the activation of the UPR^mt^ was first discovered in *C. elegans* and is nicely summarized by Suhm and Ott (2017). In 2003, an siRNA library screen, aiming to identify proteins that are affecting the lifespan of worms, was performed (Lee et al., 2003). Interestingly, some of the identified proteins (e.g., MRPS5) are involved in mitochondrial gene expression and depletion leads to reduced ATP levels and a disruption of the mitochondrial network. However, worms also exhibited increased resistance against stress inducing reagents, such as H_2_O_2_ (Lemieux et al., 2001; Lee et al., 2003). Later, experiments in mice and *C. elegans*, discovered that knockdown of the mitoribosomal subunit, MRPS5, leads to mitonuclear protein imbalance and UPR^mt^ activation (Houtkooper et al., 2013). Remarkably, the life span of these animals was increased (Houtkooper et al., 2013). The observed molecular phenotypes reflected an activation of the UPR^mt^, one of the hallmarks being a reduction in the synthesis of respiratory chain subunits, attributed to the presence of the peptide-transporter HAF-1 (Haynes et al., 2010).

**Figure 2:**
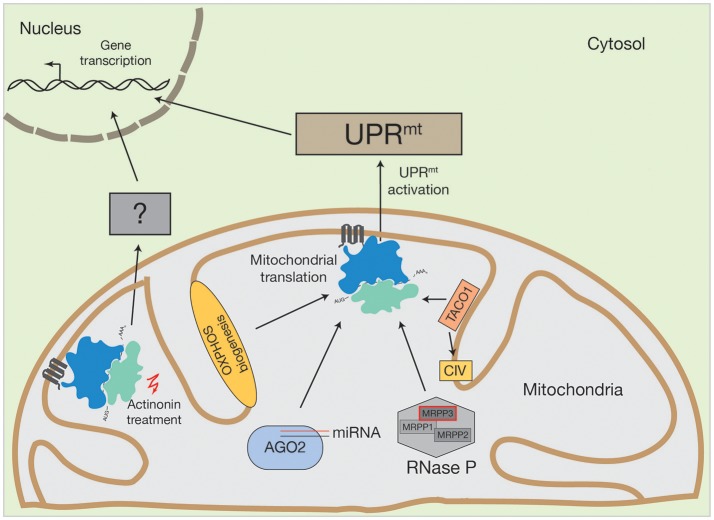
Activation of human UPR^mt^ by altered mitochondrial translation. Conceivable options to initiate UPR^mt^ by mitochondrial translation could be OXPHOS biogenesis, mitochondrial miRNA molecules in association with AGO2, PNPase-mediated mRNA processing or translational regulators, such as TACO1 (specific for cytochrome *c* oxidase (CIV)). Inhibition of mitochondrial translation with different drugs also initiate the UPR^mt^. However, actinonin treatment can also provoke an alternative stress response.

The same UPR^mt^ activation was observed by inhibiting mitochondrial translation with mitochondrial translation inhibitors, such as doxycycline or chloramphenicol (Houtkooper et al., 2013). Surprisingly, the inhibition of mitochondrial translation with different pharmacological agents does not always lead to the same consequences in nuclear gene expression. For example, upon treatment of human cells with actinonin, a peptide deformylase inhibitor that disrupts mitochondrial translation by stalling mitoribosomes and degrading mitoribosomal proteins, there was a decrease in mitochondrial mRNA and rRNA levels (Richter et al., 2013; Figure 2). This was not observed upon mitochondrial translation inhibition by other structurally diverse antibiotics, such as doxycycline or chloramphenicol (Battersby and Richter, 2013; Richter et al., 2013). The stalling of mitoribosomes by actinonin provokes an accumulation of mitochondrial translation products within the inner membrane, ultimately resulting in oxidative stress and finally, loss of membrane potential (Richter et al., 2013).

Treatment with these different classes of mitochondrial translation inhibitors suggests that actinonin activates an alternative pathway to the UPR^mt^, due to two main observations. First, the inhibition of mitochondrial translation with chloramphenicol did not show proliferation defects and second, alterations in the gene expression profile of actinonin treated cells were not entirely comparable to those previously described during UPR^mt^ induction, one of the main differences being that mitochondrial ribosomal proteins were not upregulated (Battersby and Richter, 2013; Richter et al., 2013). Hence, it was suggested that actinonin treatment leads to the production of abnormal mitochondrial translation products, that cause inner membrane permeabiliation and mitochondrial fragmentation, thereby activating a retrograde signaling pathway independent of the UPR^mt^ (Battersby and Richter, 2013; Richter et al., 2013; Suomalainen and Battersby, 2017). The molecular nature of this retrograde signal is unknown. However, evidence exists that the processing of the OPA1 protein, which is involved in mitochondrial fusion, is required, since OPA1 processing was altered upon actinonin treatment (Richter et al., 2013).

## How do defects in mitochondrial translation initiate mitochondrial stress responses?

Mammalian mitochondrial gene expression differs to that of the yeast system in various aspects (Meisinger et al., 2008; Richter-Dennerlein et al., 2015; Ott et al., 2016; Timón-Gómez et al., 2017). For example, yeast mitochondrial DNA (mtDNA) contains introns that are missing in higher eukaryotes (Foury et al., 1998). Especially the use of mouse models has significantly contributed to our understanding of mammalian mitochondrial gene expression (Trifunovic et al., 2004; Park et al., 2007; Metodiev et al., 2009; Cámara et al., 2011; Almajan et al., 2012; Gustafsson et al., 2016). Mammalian mtDNA has a size of ~16 kb, contains no significant introns and two noncoding regions; the D-loop, which harbors the origin of heavy-strand (H-strand) replication and both strands transcription, and a second smaller 30 nt region in which resides the origin of replication of the light-strand (L-strand) (for a comprehensive overview see Gustafsson et al., 2016). Human mtDNA encodes for two rRNAs, 22 tRNAs, and 13 proteins. The latter are all essential components of the OXPHOS machinery. Gene expression is initiated by the generation of two polycistronic transcripts, originating from the light- or heavy-strand promoter respectively and further processed into individual RNA species (Ott et al., 2016). Mammalian mtRNAs do not contain significant 5′-UTRs. Although two mammalian translation initiation factors have been described [mtIF2 (Overman et al., 2003) and mtIF3 (Christian and Spremulli, 2009)] a translation regulation system involving 5′-UTR RNA-binding translational activators that are specific for each mRNA, such as it occurs in yeast, is largely missing (Meisinger et al., 2008; Richter-Dennerlein et al., 2015; Gustafsson et al., 2016; Ott et al., 2016; Timón-Gómez et al., 2017). To this end, it is not clear how mitochondrial translation is regulated at an mRNA-specific level in mammals. However, recent studies in higher eukaryotes are now providing an insight and evidence is emerging that mitochondrial stress response pathways are closely linked to mitochondrial translation regulation.

### Initiation of mitochondrial signaling through microRNAs

MicroRNAs (miRNA) are non-coding RNA molecules, usually 18–22 nucleotides in length, that are involved in post-transcriptional gene expression (Bartel, 2004; Bartel and Chen, 2004). These miRNAs are encoded in the nuclear genome, synthesized as pre-miRNAs and pre-matured within the nucleus, prior to their export into the cytosol (Bartel and Chen, 2004). Cytosolic miRNAs assemble into the DICER-complex, where they are further processed, and afterwards engage with the AGO2 protein to form the active mRNA-regulating RNA-induced silencing complex (RISC) (Bartel and Chen, 2004). The RISC complex can bind to its target mRNA and induce mRNA degradation and consequently the abrogation of protein products (Bartel and Chen, 2004). For patients, miRNA applications are now considered as promising targeting strategies against human diseases, among them are cancer (Gabra and Salmena, 2017; Shirafkan et al., 2017), Parkinson's disease (Arshad et al., 2017) and cardiac perturbations (Chen et al., 2017). Cytosolic miRNAs have been linked to mitochondrial function, too. As reviewed by Zhao et al. (2017), cytosolic miRNAs modulate mitochondrial fission and fusion processes, they are involved in oxidative stress and they play major roles in mitochondrial initiated apoptotic pathways. Interestingly, miRNA molecules and the AGO2 protein have also been found inside mitochondria (Zhang et al., 2014) (Figure 2). Until now, the AGO2 protein is the only component of the cytosolic RISC complex that has been reported to enter mitochondria (Zhang et al., 2014; Jagannathan et al., 2015). Controversially, mitochondrial miRNAs seem to have an opposing function to that of their cytosolic counterparts. AGO2, together with miR1, increases CYTB, COX3, and ATP8 translation. AGO2 can also associate with miR-499-5p, whereby it can stimulate ND4L and ND1 translation. To date, ~150 mitochondrial miRNAs have been described (Bandiera et al., 2013; Geiger and Dalgaard, 2017). However, it has been supposed that even more are targeted to mitochondria that have diverse, yet unknown, functions (Bandiera et al., 2013; Geiger and Dalgaard, 2017).

A conceivable function of miRNAs is their involvement in cellular signaling cascades and stress sensing situations. This hypothesis is supported by the fact that miR-1 is induced during the differentiation of myoblasts to myotubes (Zhang et al., 2014). Hence, mitochondrial miRNAs have the potential to directly influence mitochondrial translation, depending on the physiological state of the cell. An alternative hypothesis could be that mitochondrial miRNAs play crucial roles during mitochondrial stress via UPR^mt^ or UPR^am^ activation. Nevertheless, the exact mechanism of how mitochondrial miRNAs regulate mitochondrial gene expression is an ongoing field of research.

### Mitochondrial RNA processing is regulated by the UPR^mt^

The human mitochondrial transcription process generates one short (containing two rRNAs) and two long mRNA and tRNA encoding polycistronic mRNA units that are further processed and modified to mature RNA molecules (Montoya et al., 1983). Human mitochondrial RNA maturation represents a multilayer system that involves several RNases and RNA modifying enzymes (Temperley et al., 2010; Rorbach and Minczuk, 2012; Bruni et al., 2017). However, a unique feature of the mammalian mRNA and tRNA encoding transcript is the distribution of the tRNA genes, which mostly flank the mRNA genes (Ojala et al., 1981). This situation requires an individual release of each tRNA from the polycistron, a process that is facilitated by ELAC2 (contains RNase Z activity and processes the 3′ ends of tRNA) and the RNase P complex (Rossmanith, 2012). The RNase P complex is composed of three subunits (MRPP1, MRPP2, and MRPP3) and matures tRNA molecules at the 5′ region. Mutations in ELAC2 cause an accumulation of mtRNA precursors and impaired mitochondrial translation and have been linked to hypertrophic cardiomyopathy (Haack et al., 2013). Interestingly, MRPP3 has been described as a target of the UPR^mt^ (Münch and Harper, 2016) (Figure 2). During UPR^mt^ activation, the stress dependent induction of the LON protease increases MRPP3 turnover, thereby reducing levels of MRPP3 (Münch and Harper, 2016). Consequently, mitochondrial RNA precursors accumulate, which concomitantly leads to impaired mitochondrial translation (Metodiev et al., 2016). Hence, it is tempting to speculate that mitochondrial mRNA processing, facilitated by the RNase P complex, is integrated into, or promotes, mitochondrial dependent stress response pathways.

### Can oxphos assembly defects initiate a mitochondrial stress response?

The mitochondrial OXPHOS machinery is composed of nuclear- and mitochondrial encoded proteins. Since the accumulation of OXPHOS sub-complexes within the inner mitochondrial membrane leads to increased ROS production and subsequent oxidative stress, the supply of subunits from both genetic systems needs to be balanced (Richter-Dennerlein et al., 2015; Dennerlein et al., 2017; Wasilewski et al., 2017). In yeast, mitochondrial protein synthesis is modulated by translational activators that mainly bind specific RNA molecules at defined positions (Mick et al., 2011; Soto et al., 2012; Herrmann et al., 2013; Kehrein et al., 2013; Dennerlein et al., 2017). In contrast, human mitochondria largely lack translational activators. One exception is TACO1, a translational regulator of the cytochrome *c* oxidase core subunit COX1 (Figure 2). TACO1 is a soluble protein that resides within the mitochondrial matrix. Its loss causes cytochrome *c* oxidase deficiency and has been implicated in Leigh syndrome (Weraarpachai et al., 2009). Interestingly, TACO1 interacts directly with COX1 mRNA and the mitochondrial ribosome (Richman et al., 2016). Consequently, mutations or loss of TACO1 lead to a reduction in COX1 synthesis (Weraarpachai et al., 2009). It is unknown which cellular stress responses are initiated under such conditions. However, since inhibition of mitochondrial translation can provoke UPR^mt^ activation (as described above), it is conceivable that functional loss of TACO1 could also activate UPR^mt^ signaling pathways.

Richter-Dennerlein et al. (2016) defined three COX1 translation ribosome-nascent chain complexes that contain C12ORF62 (COX14) and MITRAC12 (COA3). Mutations in either protein have been found in patients with cytochrome *c* oxidase deficiency that results from a reduction in COX1 translation (Mick et al., 2012; Szklarczyk et al., 2012; Weraarpachai et al., 2012; Ostergaard et al., 2015). Interestingly, siRNA mediated depletion of C12ORF62 caused a block in COX1 translation, which was released when COX4, the first nuclear-encoded structural cytochrome *c* oxidase subunit, associated with COX1 (Richter-Dennerlein et al., 2016). Hence, mitochondrial ribosomes are able to adapt mitochondrial translation according to the availability of nuclear-encoded subunits. Is this translational plasticity restricted to COX1 in human mitochondria? To date, we lack clear experimental data to answer this question. However, ribosome profiling data revealed that all human mitochondrial mRNAs are present at defined hotspots during translation (Rooijers et al., 2013). Hence, it can be speculated that the translation and assembly of other mitochondrial-encoded proteins also depends on the supply of nuclear-encoded proteins from the cytosol.

As described above, a block during mitochondrial translation leads to the activation of stress response pathways within mitochondria, which can instigate the UPR^mt^ pathway. The induction of these stress response pathways can also be considered as “checkpoints” for mitochondrial fitness and functionality. Hence, the accumulation of partially translated COX1, stalled in intermediates associated with the mitochondrial ribosome, could potentially initiate the UPR^mt^. This scenario would directly link mitochondrial OXPHOS assembly, to mitochondrial translation and UPR^mt^ activation, but this hypothesis requires further research.

## Conclusions

The synchronization of mitochondrial translation and OXPHOS assembly with cell metabolic demands is vital for homeostasis. As studies continue to uncover the mechanisms of mitochondrial translation regulation in mammals it becomes increasingly apparent that there exists an important route of communication from mitochondria to the nucleus. Very little is known about these retrograde signaling pathways. The UPR^mt^, originally identified as a pathway that recognizes internal mitochondrial imbalances, has now been implicated in mitochondrial translation defects. Although evidence is still sparse, it is plausible that due to the tight synchronization between the translation and assembly of OXPHOS components, the UPR^mt^ also senses defects in OXPHOS biogenesis.

These findings drive several key questions; what are the molecular cascades that link mitochondrial translation defects to the UPR^mt^? Which alternative signaling pathways exist? What is the role of miRNAs in mitochondrial stress signaling? And how are defects in OXPHOS assembly signaled? A number of mitochondrial diseases, that result from either mutations in mtDNA, or in nuclear-encoded mitochondrial genes, cause OXPHOS defects (Suomalainen and Battersby, 2017). An elucidation of how mitochondria communicate translation and assembly defects with the nucleus is particularly imperative to understand how the cell responds in these cases and would ultimately provide novel pathways for targeted treatment.

## Author contributions

All authors listed have made a substantial, direct, and intellectual contribution to the work, and approved it for publication.

### Conflict of interest statement

The authors declare that the research was conducted in the absence of any commercial or financial relationships that could be construed as a potential conflict of interest.
